# Influence of Al_2_O_3_ nanoparticle doping on depolarization temperature, and electrical and energy harvesting properties of lead-free 0.94(Bi_0.5_Na_0.5_)TiO_3_–0.06BaTiO_3_ ceramics

**DOI:** 10.1039/d0ra04866f

**Published:** 2020-08-28

**Authors:** Pharatree Jaita, Supalak Manotham, Gobwute Rujijanagul

**Affiliations:** Department of Physics and Materials Science, Faculty of Science, Chiang Mai University Chiang Mai 50200 Thailand rujijanagul@yahoo.com; Science and Technology Research Institute, Chiang Mai University Chiang Mai 50200 Thailand; Research Center in Physics and Astronomy, Faculty of Science, Chiang Mai University Chiang Mai 50200 Thailand; Materials Science Research Center, Faculty of Science, Chiang Mai University Chiang Mai 50200 Thailand

## Abstract

In this research article, the effects of Al_2_O_3_ nanoparticles (0–1.0 mol%) on the phase formation, microstructure, dielectric, ferroelectric, piezoelectric, electric field-induced strain and energy harvesting properties of the 0.94(Bi_0.5_Na_0.5_)TiO_3_–0.06BaTiO_3_ (BNT–6BT) ceramic were investigated. All ceramics have been synthesized by a conventional mixed oxide method. The XRD and Raman spectra showed coexisting rhombohedral and tetragonal phases throughout the entire compositional range. An increase of the grain size, *T*_F–R_, *T*_m_, *ε*_max_ and *δ*_A_ values was noticeable when Al_2_O_3_ was added. Depolarization temperature (*T*_d_), which was determined by the thermally stimulated depolarization current (TSDC), tended to increase with Al_2_O_3_ content. The good ferroelectric properties (*P*_r_ = 32.64 μC cm^−2^, *E*_c_ = 30.59 kV cm^−1^) and large low-field *d*_33_ (205 pC N^−1^) values were observed for the 0.1 mol% Al_2_O_3_ ceramic. The small Al_2_O_3_ additive improved the electric field-induced strain (*S*_max_ and 
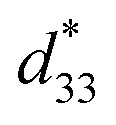
). The 1.0 mol% Al_2_O_3_ ceramic had a large piezoelectric voltage coefficient (*g*_33_ = 32.6 × 10^−3^ Vm N^−1^) and good dielectric properties (*ε*_r,max_ = 6542, *T*_d_ = 93 °C, *T*_F–R_ = 108 °C, *T*_m_ = 324 °C and *δ*_A_ = 164 K). The highest off-resonance figure of merit (FoM) for energy harvesting of 6.36 pm^2^ N^−1^ was also observed for the 1.0 mol% Al_2_O_3_ ceramic, which is suggesting that this ceramic has potential to be one of the promising lead-free piezoelectric candidates for further use in energy harvesting applications.

## Abstract 2

In this research article, the effects of Al_2_O_3_ nanoparticles (0–1.0 mol%) on the phase, microstructure, dielectric, ferroelectric, piezoelectric, electric field-induced strain and energy harvesting of the BNT–6BT ceramic were investigated.

## Graphical abstract

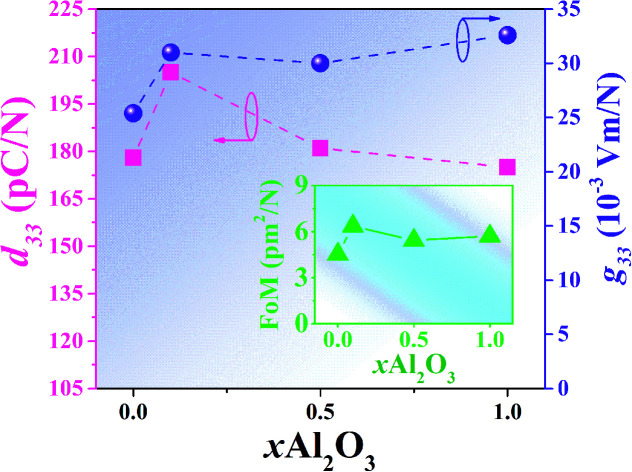

## Introduction

Piezoelectric ceramics currently in large-scale applications are still lead-based ceramic systems, which have outstanding piezoelectric properties.^1^ However, the high lead content (near 60%) in PZT electronic ceramic devices is harmful to our environment and the human body during the production and usage processes of these devices.^2^ The search for lead-free piezoelectric ceramic systems, such as Ba_0.5_Na_0.5_TiO_3_ (BNT),^1,3^, BaTiO_3_ (BT)^4^ and BiFeO_3_ (BF),^5^ has therefore attracted more efforts.

Among the candidates for lead-free systems, the Bi-based ceramic like bismuth sodium titanate Bi_0.5_Na_0.5_TiO_3_ (BNT) is regarded as the most promising system comparable to lead-based materials, and it has been extensively studied.^1,2^ BNT is one of the comprehensively studied lead-free perovskite ferroelectric materials, which exhibits a relatively high Curie temperature (*T*_C_ = 320 °C) as well as a high remanent polarization (*P*_r_ ∼ 38 μC cm^−2^).^6^ BNT has a rhombohedral perovskite structure (*R*3*c*) with Na^1+^ and Bi^3+^ distributed sequentially in A-site.^7^ However, high value of the coercive electric field (*E*_c_ > 60 kV cm^−1^) and high conductivity are two drawbacks for BNT to become an alternative replacement for PZT.^7^ A recent research by Nagata *et al.*^8^ using secondary ion mass spectrometry revealed that the difficulty in the poling treatment of the BNT ceramic is attributed to the pinning of domain movement due to Bi vaporization at high sintering temperatures above 1130 °C. This difficulty is causing limitations in the applications of the BNT. One of the possible solutions to overcome the drawbacks in the BNT ceramic, is to use a solid solution by substituting of either A or B-sites.^7^ Furthermore, researchers found that establishing the binary system of BNT–BT is an effective way to improve the electrical performances of this system.^9,10^ The (1 − *x*)(Bi_0.5_Na_0.5_)TiO_3_–*x*BaTiO_3_ or (1 − *x*)BNT–*x*BT system has been firstly studied by Takenaka *et al.*^9^ for its dielectric and piezoelectric properties. This BNT–BT system is studied from the viewpoint of a new group of lead-free piezoelectric ceramics with the morphotropic phase boundary or MPB.^9^ The MPB can be obtained from the compositional boundary between two different perovskite phases which can be achieved by controlling the ratio of each perovskite component. The origin of the high piezoelectric response in the MPB can be explained by a high of degree freedom of the polarization within the boundary which can be easily oriented by an electric field.^11^ In their work, the MPB between rhombohedral BNT and tetragonal BT phases was found at *x* = 0.06 − 0.07 with highly enhanced piezoelectric (*d*_33_ = 125 pC N^−1^, *k*_33_ = 55%) and dielectric properties (*ε*_r_ = 580, *T*_C_ = 288 °C), with the depolarization temperature (*T*_d_) between 100–130 °C. The binary system of BNT–BT with its MPB composition has superior piezoelectric properties, which means that this composition has a potential to be one of the promising lead-free piezoelectric candidates for actuator applications.^9^

Although the Bi_0.5_Na_0.5_TiO_3_-based solid solution is one of the most promising lead-free piezoelectric candidates, the depolarization temperature (*T*_d_) of these solid solutions is an obstacle for their practical applications,^12,13^*i.e.* the BNT–BT ceramic started to loss piezoelectric properties around *T*_d_ of 100 °C, the BNT–BKT ceramic has a similar polarization and started to lose those properties at 150 °C, and K_0.5_Na_0.5_NbO_3_ (KNN) ceramic shows a polymorphic phase transition (PPT) between orthorhombic and tetragonal structure at ∼200 °C, above which the piezoelectric properties degrade.^14^ Thus, many authors have tried to increase the *T*_d_ value of BNT-based ceramics so that these ceramics would also achieve high piezoelectric properties.^12,13,15^ Zhang *et al.*^12^ prepared the semiconductor/relaxor 0–3-type composites of 0.94Bi_0.5_Na_0.5_TiO_3_–0.06BaTiO_3_ : *x*ZnO (or BNT–6BT : *x*ZnO). They found that the *T*_d_ increased from 98 °C for the *x* = 0 sample to ∼150 °C for the *x* = 0.2 sample, indicating a delay of thermal depolarization. As the ZnO concentration further increased to *x* = 0.3 and *x* = 0.4, the first anomaly of dielectric constant and the sharp decrease of dielectric loss disappeared, indicating the complete elimination of thermal depolarization. Mahajan *et al.*^15^ studied the thermal depoling behavior of the BNTBT–ZnO composites. This work clearly showed the shift in depolarization temperature (*T*_d_) to a higher temperature, which was explained to be due to the intrinsic contribution of Zn ions in the BNTBT lattice. In this work, the thermal depoling behavior of BNTBT-based materials was also directly related to the transition temperature from the rhombohedral phase to the tetragonal phase in this phase transition model, which was consistent with four current peaks in their ferroelectric loops close to the depoling temperature. Takagi *et al.*^16^ also found that the quenching was an effective increase of the *T*_d_ value for the lead-free (Bi_0.5_Na_0.5_)TiO_3_–(Bi_0.5_Li_0.5_)TiO_3_–(Bi_0.5_K_0.5_)TiO_3_ system without the deterioration of its piezoelectric properties. The increase in *T*_d_ was strongly correlated with the lattice distortion. Verma *et al.*^17^ also found that the addition of vanadium (V^5+^) increased the *T*_d_ of the 0.94(Na_0.50_Bi_0.50_)TiO_3_–0.06BaTiO_3_ or BNT–BT ceramics. The *T*_d_ was found to increase significantly in poled samples from 104 °C for the undoped sample to 150 °C for the sample with 1% vanadium substitution.

Besides these additives, some other alternative metal oxide additives should be introduced to shift up the *T*_d_ as well as to improve the electrical properties of the BNT-based ceramics. It is suggested form the work done by Yin *et al.*^13^ that Al_2_O_3_ can significantly shift up the *T*_d_ in the BNKT-based materials (Bi_0.5_(Na_0.8_K_0.2_)_0.5_TiO_3_ : Al_2_O_3_ or BNKT : Al_2_O_3_ composites). This is evidenced by the temperature dependence of the dielectric constant, ferroelectric and piezoelectric properties. In addition, the piezoelectricity of the BNKT : 0.15Al_2_O_3_ ceramic remained stable at a high temperature (∼210 °C).^13^ However, the effects of the Al_2_O_3_ on the *T*_d_ for the BNT–BT ceramic have not been researched, to our knowledge. Furthermore, the effects of the Al_2_O_3_ additive on electrical properties such as the energy harvesting properties of the BNT–6BT ceramics are scarcely known. In the present work, the lead-free 0.94(Bi_0.5_Na_0.5_)TiO_3_–0.06BaTiO_3_ (BNT–6BT, at the MPB composition) was selected due to its showing higher piezoelectric and ferroelectric properties. The Al_2_O_3_ nanoparticle powder was selected as the additive because it is expected that when some Al^3+^ ions enter into the perovskite lattice to replace the Ti^4+^ ions at Bi-site, the imbalance in ion valence can lead to the creation of oxygen vacancies, which, in turn, enhances the transfer of mass and energy between reactants, with the end result of an increases in grain size.^18,19^ It is well known for ferroelectric ceramics that have larger grains, domain motion is easier which results in an increase in its ferroelectric performance.^20^ Moreover, when Al^3+^ occupies the Bi-site of Ti^4+^, it will create oxygen vacancy and produce lattice deformation. This may make the ferroelectric domains reorientation easier during the electrical poling, and then lead to the enhancement of piezoelectric properties.^19^ It was also reported that some nanoparticle additive produced a significant difference on microstructure over the micro size additive.^21^ For example in the KNN ceramics, the nanoparticle of the ZnO additive improved their grain growth, along with decreasing the porosity, and then resulted in the enhancements of piezoelectric and dielectric properties as compared to the microparticle ZnO additive. This evidence was explained by the better modification of microstructure after the ceramics were added by the nanoparticle additive.^21^ Furthermore, our previous work indicated that the addition of Al_2_O_3_ nanoparticles in lead-based ceramics such as Pb_0.88_Sr_0.12_Zr_0.54_Ti_0.44_Sb_0.02_O_3_ (PSZST) ceramics also improved their densification and electrical properties such as dielectric constant and polarization value.^22^ Thus, this research article aimed to fabricate the binary system of BNT–6BT/*x*Al_2_O_3_. The role of Al_2_O_3_ nanoparticle concentration on densification, phase evolution, microstructure, electrical properties (dielectric, ferroelectric, piezoelectric, electric field-induced strain behavior), depolarization temperature and energy harvesting properties of the BNT–6BT ceramic were investigated and discussed in details.

## Experimental

The conventional mixed oxide technique was applied to synthesize the powder of 0.94(Bi_0.5_Na_0.5_)TiO_3_–0.06BaTiO_3_/*x*Al_2_O_3_ nanoparticles, referred to as BNT–6BT/*x*Al_2_O_3_ ceramics. Reagent-grade powders of Bi_2_O_3_, Na_2_CO_3_, BaCO_3_, TiO_2_, Al_2_O_3_ nanoparticles were used as the starting raw materials. All carbonate powders were first dried at 120 °C for 24 h in order to remove any moisture. The raw materials of BNBT were stoichiometrically weighed and mixed by ball milling in 99.9% ethanol for 24 h and the slurry was dried using an oven. Dried BNBT powders were separately calcined in a closed Al_2_O_3_ crucible at 900 °C for 2 h. After that, the Al_2_O_3_ nanoparticles were added to calcined BNT–6BT powder in different ratios (0, 0.1, 0.5 and 1.0 mol%) and then milled for 24 h in ethanol. After drying and sieving the powders, they were granulated by adding a few drops of 4 wt% PVA as a binder and then pressed into disks 10 mm in diameter and about 1.2 mm in thickness. Finally, the pellets were placed in a sealed alumina crucible and covered with a powder of the same composition before being sintered at 1125 °C for 2 h dwell time with a heating/cooling rate of 5 °C min^−1^. Bulk density was determined by the Archimedes' method. An X-ray diffractometer ((XRD), PANalytical, X′ Pert Pro MPD) was used to study the phase evolution of all ceramics. Raman spectra were obtained by Raman spectroscopy. A scanning electron microscope (SEM, JEOL JSM-6335F) was used to study microstructural features of the ceramics. The grain size of the ceramics was measured by using the linear intercept method. Before electrical measurements were performed, all samples were polished into a parallel surface with 1 mm thickness. Silver paste was painted onto both sides of the sample. Then the samples were heated at 700 °C for 15 min to form electrodes. Dielectric properties as a function of temperature (25–500 °C) were determined using an LCR-meter (HP model 4192A) at frequencies ranging from 1 to 1000 kHz. The ferroelectric properties were investigated by a Radiant Precision ferroelectric tester both at room temperature (RT) and high temperatures (HT) of 25–150 °C. An AC electric field of 50 kV cm^−1^ at a frequency of 1 Hz was utilized in the hysteresis measurement. Remanent polarization (*P*_r_), maximum polarization (*P*_max_), and coercive field (*E*_c_) values were determined from the hysteresis loops. Strain–electric field (*S*–*E*) data at RT were obtained by using an optical displacement sensor (Fotonic Sensor model MTI-2100) injunction with a Radiant ferroelectric test system. A maximum electric field of 50 kV cm^−1^ and a frequency of 0.1 Hz were used to measure the bipolar strain curve. The maximum strain (*S*_max_) and the negative strain (*S*_neg_) values were calculated from the bipolar curve. The normalized strain coefficient 
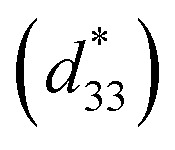
 was also determined following: 
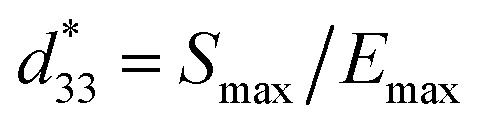
. For piezoelectric characterization, the ceramics were poled in silicone oil under a DC electric field of 3 kV mm^−1^ for 15 min. The low-field piezoelectric coefficient (*d*_33_) was recorded from the 1 day aged samples using a *d*_33_ meter (KCF technologies, S5865) at a frequency of 50 Hz. The piezoelectric voltage constant (*g*_33_) and the off-resonance figure of merit (FoM) for energy harvesting were also calculated.

## Results and discussion

### Densification, phase formation and microstructure

After sintering the pellet-shaped samples at various temperatures (1100–1175 °C), it was found that the optimum sintering temperature of the BNT–6BT/*x*Al_2_O_3_ ceramics was 1125 °C at which all samples had densities ranging between 5.77 and 5.86 g cm^−3^. Based on the density data at the optimum sintering temperature of 1125 °C, the data clearly showed that the variation of composition had a significant influence on the sample density. The addition of Al_2_O_3_ nanoparticles into the BNT–6BT ceramic caused a slight decrease in the sample's density. The reason for this slight decrease in the samples' density was probably due to the fact that Al_2_O_3_ had a lower density (∼3.59 g cm^−3^)^23^ than the BNT–6BT ceramic (∼5.841 g cm^−3^).^24^

**Fig. 1:**
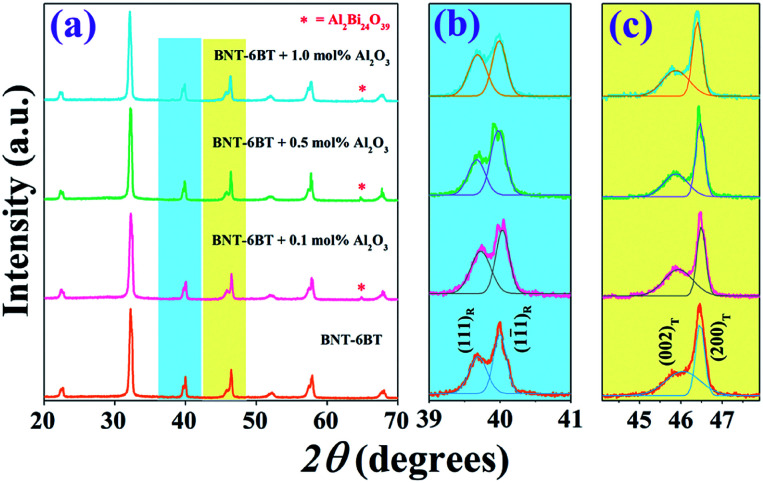
X-ray diffraction patterns of the BNT–6BT/*x*Al_2_O_3_ ceramics where (a) 2*θ* = 20–80°, (b) 2*θ* = 39–41°, and (c) 2*θ* = 45–47.5°.

XRD patterns of the BNT–6BT/*x*Al_2_O_3_ ceramics at room temperature (RT) are shown in Fig. 1(a), where 2*θ* = 20–70°. It was found that the pure BNT–6BT ceramic had a single perovskite structure with a mix of rhombohedral and tetragonal phases. This was evidenced by a slight splitting of (111)_*R*_/(11̄1)_*R*_ rhombohedral peaks at 2*θ* = 39–41° and (002)_*T*_/(200)_*T*_ tetragonal peaks at 2*θ* = 45–48°, as seen in Fig. 1(b) and (c), respectively. This result is consistent with the previous work reported by Takenaka *et al.*^9^ who found a relatively broad morphotropic phase boundary (MPB) region between rhombohedral BNT and tetragonal BT phases in the range of *x* = 0.06–0.07. Chen *et al.*^25^ also found that the BNT–6BT ceramic at MPB region had a mixture of rhombohedral and tetragonal phases with the splitting of (111)_*R*_/(11̄1)_*R*_ around 40° and (002)_*T*_/(200)_*T*_ peak around 46°, respectively. For the modified-samples, the coexistence of the mixed rhombohedral and tetragonal phases was also obtained. This can be evidenced by, the existence of the splitting (002)_*T*_/(200)_*T*_ tetragonal peaks, and (111)_*R*_/(11̄1)_*R*_ rhombohedral peak, thus indicating that all ceramics showed coexisting rhombohedral and tetragonal phases throughout the entire compositional range. Based on Table 1, the tetragonality (*c*/*a*) value decreased from 1.0121 for the pure BNT–6BT ceramic to 1.0115 for the 1.0 mol% Al_2_O_3_ ceramic. These results may be due to apart of Al ions from the additive having replaced Ti ions (ionic radius of Ti^4+^ = 0.605 Å and Al^3+^ = 0.535 Å).^26^ This produced a slight distortion of the lattice and clearly indicated that the addition of Al_2_O_3_ increased the lattice anisotropy of the BNT–6BT systems. However, a secondary phase of aluminum bismuth oxide, Al_2_Bi_24_O_39_ (ICDD file no. 00-042-0184) was observed at 2*θ* ∼ 64.8° with an increase of Al_2_O_3_ content. A similar result was observed in the BNKT–Al_2_O_3_ system, of which the secondary phase of Al_2_Bi_24_O_39_ exhibited more with an increase in the additive.^13^

**Table 1:** Physical, phase, microstructure and dielectric properties of the BNT–6BT/*x*Al_2_O_3_ ceramics

*x*	Density (g cm^−3^)	Grain size (μm)	*c*/*a*	*T*_d_ (°C)	*T*_F–R_ (°C)	*T*_m_ (°C)	*ε*_max_	tan *δ*	*δ*_A_ (K)
0	5.86	1.81	1.0121	86	93	293	5848	0.0563	89
0.1	5.79	1.92	1.0119	87	96	294	5768	0.0795	107
0.5	5.79	2.29	1.0117	91	99	295	6064	0.1014	126
1.0	5.77	3.12	1.0115	93	108	324	6542	0.2048	164

**Fig. 2:**
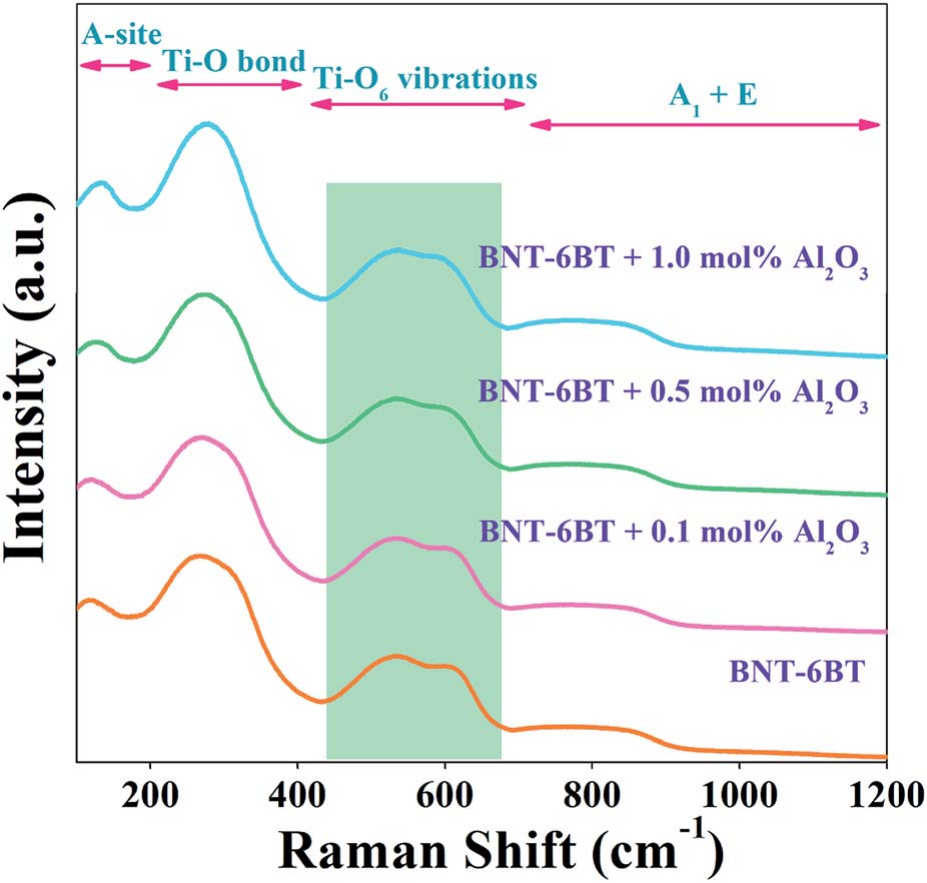
Raman spectra of the BNT–6BT/*x*Al_2_O_3_ ceramics at RT.

Raman spectroscopy is an effective tool for identifying the structural modification and lattice vibrations of the crystals. In order to identify the functional groups in the present ceramics, we carried room temperature Raman measurements on the smooth surface of the BNT–6BT/*x*Al_2_O_3_ ceramics in 100–1200 cm^−1^. Raman modes mainly occur due to the internal vibrations of lattice point in octahedral/tetrahedral sites. Here, the Raman spectra of the BNT–6BT/*x*Al_2_O_3_ ceramics at RT are presented in Fig. 2. The obtained modes of Raman spectra are similar to these connected to the BNT-based ceramics that were reported in the literature earlier.^1,27,28^ Four main regions were detected in the Raman spectra: (1) the mode below 200 cm^−1^ related to A-site vibrations,^29^ including Bi^3+^, Na^+^ and Ba^2+^; (2) the peak at 260 cm^−1^ associated with the Ti–O bond which is in agreement with the peak of 267–270 cm^−1^ previously reported by Baskar *et al.*;^30,31^ (3) the high-frequency region from 450 to 700 cm^−1^ related to TiO_6_ vibrations, namely the breathing and stretching modes of the oxygen octahedral,^27^ and (4) the fourth region where a vibration range higher than 700 cm^−1^ was associated with the superposition of vibration A_1_ (longitudinal optical) and E (longitudinal optical) overlapping bands.^27,29^ It is obvious that a special splitting of two bands was observed in between 450 and 700 cm^−1^ for all compositions, which indicates the coexistence of a dual phase between rhombohedral and tetragonal, thus this confirms the existence of MPB in the present ceramics.^18^ Therefore, this Raman result in the present work agrees with the XRD result, which confirms the coexistence of the rhombohedral and tetragonal phases in the entire composition range.^18^

**Fig. 3:**
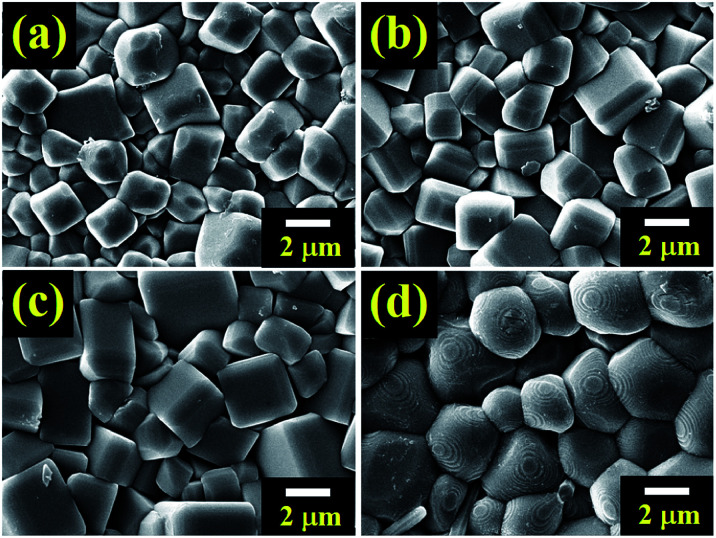
FE-SEM micrographs of as sintered surfaces of the BNT–6BT/*x*Al_2_O_3_ ceramics where (a) *x* = 0, (b) *x* = 0.1, (c) *x* = 0.5, and (d) *x* = 1.0 mol%.

The microstructures of the BNT–6BT/*x*Al_2_O_3_ ceramics are exhibited in Fig. 3. The average grain size was calculated by the linear intercept method. SEM observation confirmed that all ceramic samples displayed a highly dense and compact microstructure without a trace of porosity. Sharp grain boundaries were observed from the SEM images. The pure BNT–6BT ceramic showed a dense and homogeneous microstructure with regular cubic-like grains. The addition of Al_2_O_3_ allowed a more equiaxed grain shape. The average grain size increased slightly from 1.81 μm for the pure BNT–6BT ceramic by approximately 2 times to 3.12 μm for the 1.0 mol% Al_2_O_3_ ceramic, as listed in Table 1. This implies that Al_2_O_3_ can promote grain growth for the BNT–6BT ceramic. The Al ions, mostly with +3 valence, enter the perovskite lattice to replace the Ti^4+^ site. Therefore, the imbalance in ion valence leads to the creation of oxygen vacancies,^18^ which enhance the transfer of mass and energy between reactants, thus improving sintering behaviour and inducing an increase in grain size.^18^ A similar observation was made in the BNKSTT–100*x*Li system when the introduction of Li^+^ ions into the A-site of Bi_0.5_(Na_0.4_K_0.1_)_0.96_Sr_0.04_Ti_0.975_Ta_0.025_O_3_ and Li^+^ enhance mass transfer and promoted grain growth in the BNKSTT ceramics.^32^ Lee *et al.*^19^ also found that the ZnO promoted grain growth within the BNT ceramic. They found that the possible reasons for the grain growth was the fact that Zn^2+^ entered into the B-site of the perovskite structure to substitute for Ti^4+^ due to their matching radii. To maintain overall electrical neutrality, oxygen vacancies are created. As is generally recognized, the presence of oxygen vacancies is beneficial to mass transport during sintering. This is assumed to be responsible for the promoted grain growth as the content of ZnO increases in their mixture.^19^

### Dielectric properties and phase transition

**Fig. 4:**
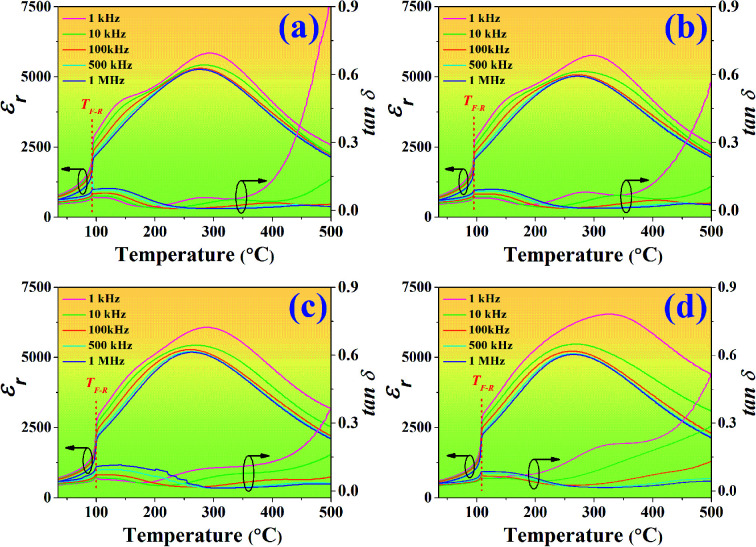
Temperature dependence on dielectric constant (*ε*_r_) and dielectric loss (tan *δ*) of the poled BNT–6BT/*x*Al_2_O_3_ ceramics and measured at various frequencies from 1–1000 kHz.

Temperature dependence on dielectric constant (*ε*_r_) and dielectric loss (tan *δ*) of the poled BNT–6BT/*x*Al_2_O_3_ ceramics measured at various frequencies from 1–1000 kHz are presented in Fig. 4, and the related dielectric values are listed in Table 1. The dielectric permittivity curves of all compositions present two anomalous dielectric peaks. These two peak anomalies intrinsically come from pure BNT in which the coexistence of *R*3*c* and *P*4*bm* polar nanoregions (PNRs) has been reported, which can be reversibly transformed into each other over a wide temperature range.^33^ The peak position around higher temperature is defined as *T*_m_, where the dielectric constant reaches the maximum value (*ε*_max_).^32^ The peaks of lower temperatures are called ferroelectric to relaxor transition point (*T*_F–R_), which denotes the transformation from the long-range ferroelectric state (FE) to the relaxor state.^1,3,34,35^ Such phenomena have also been reported in other BNT-based ceramics.^1,3,6,34–36^ Sharp *T*_F–R_ peaks can be clearly seen in the pure BNT–6BT and the doped-ceramics. In this work, the *T*_F–R_ for the pure BNT–6BT ceramic was 93 °C which was close to 86 °C the value observed earlier by Gong *et al.*^6^ The *T*_F–R_ was obviously composition-dependent and was found to increase with increasing Al_2_O_3_ content. This value reached the maximum value of 108 °C for the 1.0 mol% Al_2_O_3_ ceramic (enhanced from the based composition by 15 °C). The pure BNT–6BT ceramic had *T*_m_ and *ε*_max_ value of 293 °C and 5848, respectively. The *T*_m_ value in this work was close to *T*_m_ = 300 °C of 0.94(Bi_0.5_Na_0.5_)TiO_3_–0.06BaTiO_3_ ceramic which was observed previously by Yan *et al.*^1^ The *T*_m_ and *ε*_max_ values also increased with increasing Al_2_O_3_ content. A reason for the increase in *ε*_max_ may be due to the Al^3+^ ions that can be replaced by Ti^4+^ ions on the B-site of the perovskite ABO_3_ structure within the BNT–6BT ceramic. This leads to a rise in the local deformation of the unit cell and a significant increase in its permittivity performance.^19^ The tan *δ* value also follows a similar trend to that of the *ε*_max_ value. The tan *δ* increased with increasing Al_2_O_3_ content. Besides, the dielectric peaks at *T*_m_ were suppressed and gradually broadened with increasing Al_2_O_3_ content, suggesting an enhanced diffuse phase transition behaviour of this system.^37,38^

To quantify the degree of diffuseness at the phase transition at *T*_m_, a diffuseness parameter (*δ*_A_) was calculated by using the modified Curie–Weiss law, as the following equation.^39,40^1
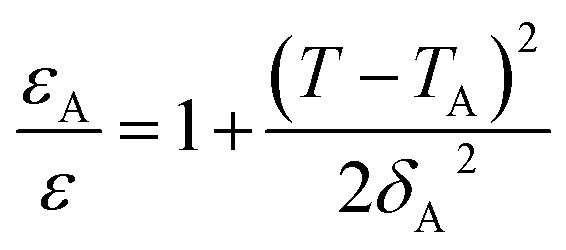
where *T* is the absolute temperature, *T*_A_ and *ε*_A_ are extrapolate parameters, *ε* is the dielectric constant, and *δ*_A_ is the diffuseness parameter. The *δ*_A_ value was calculated from a plot of ln(*ε*_A_/*ε* − 1) *versus* the ln(*T* − *T*_A_)^2^, and the value is listed in Table 1. It can be seen that the *δ*_A_ increased with increasing Al_2_O_3_ content and reached a maximum value of 164 K for the 1.0 mol% Al_2_O_3_ ceramic. This confirms that the diffuse phase transition is stronger for the compositions with a higher Al_2_O_3_ content.

**Fig. 5:**
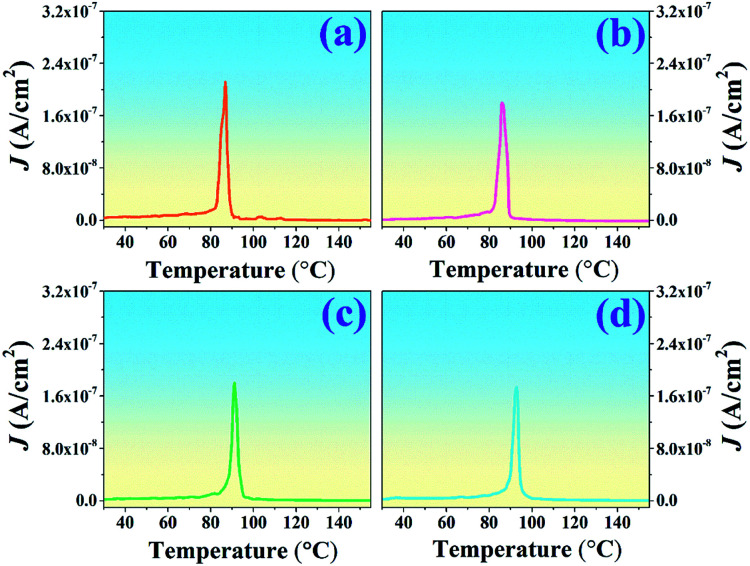
Plots of current density–temperature (*J*–*T*) from the TSDC technique of the BNT–6BT/*x*Al_2_O_3_ ceramics.

Several methods to determine the thermal depolarization temperature (*T*_d_) can be found in the literatures^14,41–44^ including the thermally stimulated depolarization current (TSDC), temperature dependent *in situ d*_33_, dielectric permittivity and dielectric loss, piezoelectric resonance measurements, and high-temperature *in situ* XRD.^14^ However, the TSDC method is one of the most popular techniques. Thus, the TSDC technique was employed in this work. The current density–temperature (*J*–*T*) curves from the TSDC technique for all studied ceramics are displayed in Fig. 5. It should be noted that *T*_d_ tended to be gradually deferred with increasing Al_2_O_3_ content. As further seen in Table 1, the *T*_d_ increased from 86 °C for the pure BNT–6BT ceramic to the maximum value of 93 °C for the 1.0 mol% Al_2_O_3_ ceramic. To explain the increase of the *T*_d_ value, many models have been proposed.^13,15,44^ In the case of the Bi_0.5_(Na_0.8_K_0.2_)_0.5_TiO_3_ : Al_2_O_3_ (BNKT : Al_2_O_3_) composites which was reported by Yin *et al.*,^13^ they found that the *T*_d_ is deferred to the higher temperatures (from 116 °C to 227 °C) with increasing Al_2_O_3_ contents, as evidenced by the temperature dependence of dielectric, ferroelectric and piezoelectric properties. In addition, the piezoelectricity of their sample (BNKT : 0.15Al_2_O_3_) remained stable at a high temperature (∼210 °C). It can be seen that Al_2_O_3_ significantly enhanced the thermal depolarization temperature. This was proposed to be due to the thermal deviatoric stress from the coefficients of thermal expansion (CTE) discrepancies between Al_2_O_3_ and BNKT matrix, which provides a stronger stabilization force than the ions diffusion-induced destabilization force, resulting in the ultimate deferred thermal depolarization and the significantly increased *T*_d_ values.^13^ Mahajan *et al.*^15^ also studied thermal depoling behavior of the BNTBT–ZnO composites. They also suggested that the shift up of *T*_d_ in their samples is due to Zn ions being diffused into the BNTBT lattices and occupying the B-sites, thereby stabilizing ferroelectric ordering. Li *et al.*^44^ reported that the doping of Zn^2+^ into Bi_0.5_Na_0.5_TiO_3_–6BaTiO_3_ (BNT–6BT) delayed the crossover from nonergodic to ergodic states, and the thermal depolarization temperature *T*_d_ was delayed from 85 °C for the pure sample to 120 °C for the BNT–6BT + 6% Zn^2+^ sample, as confirmed by temperature-dependent dielectric and ferroelectric measurements. They suggested that the variation of the *T*_d_ could be ascribed to the reformation of the long-range ferroelectric order due to the large ionic polarizability of Zn^2+^. The high ionic polarizability of Zn^2+^ can result in a large dipole moment of BO_6_ octahedra, thus strengthening the coherence of neighboring dipoles and suppressing the ferroelectric–relaxor transition. These results improve our understanding on the thermal depolarization of Bi_0.5_Na_0.5_TiO_3_-based ferroelectrics.^44^ In the present work for BNT–6BT/*x*Al_2_O_3_ ceramics, it is believed that the shift in the depolarization temperature is due to the intrinsic contribution of the Al ion in the BNT–6BT lattices, which is the reason for the increasing of *T*_d_ value, as already suggested by many authors.

### Ferroelectric and electric field induced strain properties

**Fig. 6:**
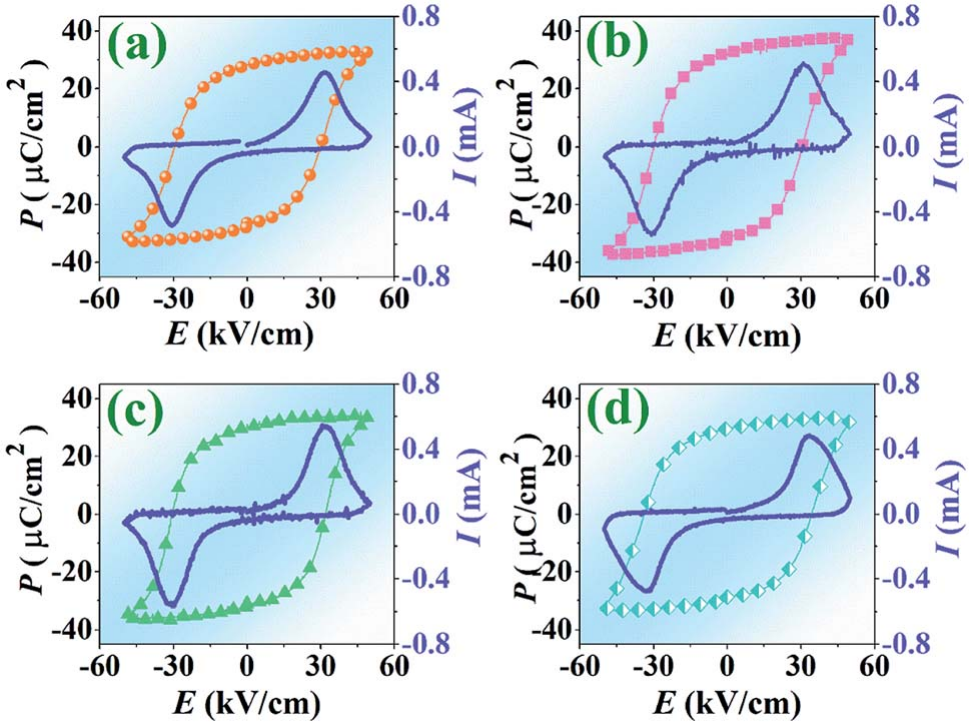
Polarization–electric field (*P*–*E*) hysteresis loop and current–electric field (*I*–*E*) of the BNT–6BT/*x*Al_2_O_3_ ceramics, measured at RT under an electric field of 50 kV cm^−1^ a frequency of 1 Hz where (a) *x* = 0, (b) *x* = 0.1, (c) *x* = 0.5, and (d) *x* = 1.0 mol%.

In this work, the polarization–electric field (*P*–*E*) hysteresis loop of the BNT–6BT/*x*Al_2_O_3_ ceramics is measured at RT under an electric field of 50 kV cm^−1^ and a frequency of 1 Hz where *x* = 0–1.0 mol% are plotted in Fig. 6. The corresponding values of remanent polarization (*P*_r_), maximum polarization (*P*_max_), and coercive field (*E*_c_) as a function of Al_2_O_3_ content are listed in Table 2. The pure BNT–6BT ceramic displayed a typical ferroelectric behavior with square-shaped *P*–*E* loops having a large maximum polarization, *P*_max_ = 32.27 μC cm^−2^, remanent polarization, *P*_r_ = 28.25 μC cm^−2^ and a relatively large coercive field, *E*_c_ = 29.95 kV cm^−1^. It should be noted that *P*_r_ and *E*_c_ values of pure BNT–6BT ceramic in this work are close to *P*_r_ (30 μC cm^−2^) and *E*_c_ (30 kV cm^−1^) values of 0.94(Bi_0.5_Na_0.5_)TiO_3_–0.06BaTiO_3_ as observed previously by Yan *et al.*^1^ Current *vs.* electric field (*I*–*E*) data are also shown in Fig. 6. Two current density peaks gradually shift to the higher field, accompanied by the slightly increased *E*_c_ value. However, the addition of Al_2_O_3_ into BNBT ceramic slightly affects the shape of the *P*–*E* loop and other ferroelectric properties. The *P*_max_ and *P*_r_ values increased with increasing Al_2_O_3_ content and reached the maximum values of 36.58 and 32.64 μC cm^−2^, respectively for the 0.1 mol% Al_2_O_3_ ceramic, then slightly decreased with further increase in the additive. The increase in grain size may be a reason for an improvement of the ferroelectric properties with an increasing *P*_r_ value. As it is known, the ferroelectric ceramics that have larger grains, domain motion is easier which results in larger *P*_r_ value.^20^ Many reports have suggested that larger grained piezoceramics have higher ferroelectric and piezoelectric properties.^45,46^ In addition, the large grains have a stronger “binding force”, which will create more irreversible electric dipole after the cancellation of the external electric field, thus leading to a greater *P*_r_ value.^47^ This result is consistent with the previous work reported by Lee *et al.*^48^ who studied the effects of Al_2_O_3_ on the ferroelectric properties of the (Na_0.5_K_0.5_)_0.925_Li_0.075_NbO_3_, NKLN lead-free piezoceramics. They found that the ferroelectric properties of this composition system mainly depend on density and grain size. With increasing Al_2_O_3_ content up to 0.5 wt%, the density and grain size increased, accompanied by an increased ferroelectric performance *i.e.* increasing of *P*_r_ value.^48^ Furthermore, the Al_2_O_3_ nanoparticles were also found to improve the densification and the electrical properties of the Pb_0.88_Sr_0.12_Zr_0.54_Ti_0.44_Sb_0.02_O_3_ (PSZST) ceramics, such as dielectric constant and polarization.^22^ Moreover for the present work, the *E*_c_ value slightly increased with increasing Al_2_O_3_ content, *i.e.* it increased from 29.95 kV cm^−1^ for the pure BNT–6BT ceramic to around 34.36 kV cm^−1^ for the 1.0 mol% Al_2_O_3_ ceramic (see Table 2). The increase in the *E*_c_ value is consistent with the previous work reported by Yin *et al.*^13^ who found that the four current density peaks of the BNKT–Al_2_O_3_ system merge into two peaks and then gradually shift to the higher field with increasing Al_2_O_3_ content, accompanied by a slightly increased coercive field *E*_c_ value.

**Table 2:** Ferroelectric, piezoelectric and energy harvesting properties of the BNT–6BT/*x*Al_2_O_3_ ceramics

*x*	*P*_max_ (μC cm^−2^)	*P*_r_ (μC cm^−2^)	*E*_c_ (kV cm^−1^)	*S*_max_ (%)	*S*_neg_ (%)	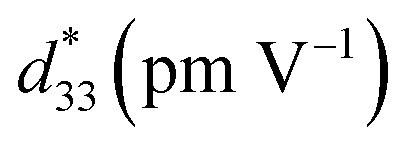	*d*_33_ (pC N^−1^)	*g*_33_ (×10^−3^ Vm N^−1^)	FoM (pm^2^ N^−1^)
0	32.27	28.25	29.95	0.21	−0.22	412	178	25.4	4.52
0.1	36.58	32.64	30.59	0.23	−0.22	466	205	31.0	6.36
0.5	33.65	30.17	31.89	0.20	−0.17	401	181	30.0	5.43
1.0	31.72	29.71	34.36	0.19	−0.16	372	175	32.6	5.71

**Fig. 7:**
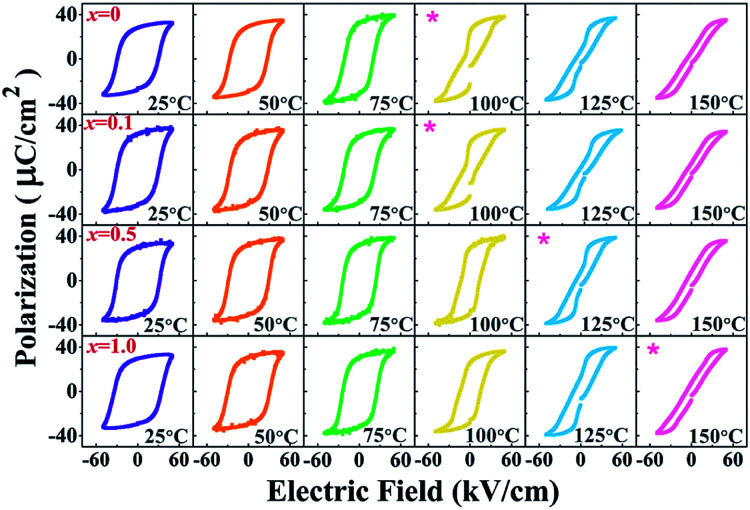
Temperature dependence on polarization–electric field (*P*–*E*) hysteresis loops of the BNT–6BT/*x*Al_2_O_3_ ceramics where *x* = 0 1.0 mol%, measured under an electric field of 50 kV cm^−1^ and a frequency of 1 Hz.

**Fig. 8:**
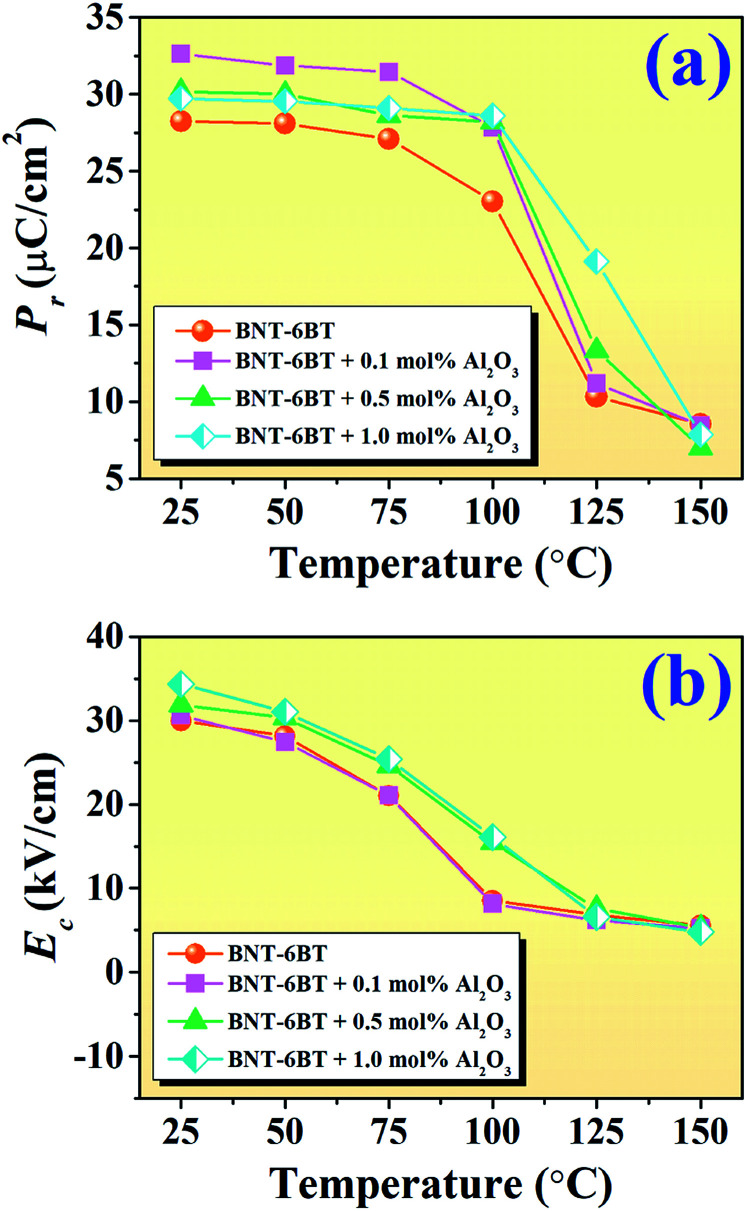
Plots of (a) *P*_r_ as a function of temperature and (b) *E*_c_ as a function of temperature of the BNT–6BT/*x*Al_2_O_3_ ceramics.

To investigate the effect of temperature on the ferroelectric properties of the BNT–6BT/*x*Al_2_O_3_ ceramics and to confirm the deferred thermal depolarization, the temperature-dependent *P*–*E* data were measured under an electric field of 50 kV cm^−1^ at 1 Hz over the temperature range of 25–150 °C, as displayed in Fig. 7. All samples exhibited well-saturated *P*–*E* loops at RT (25 °C). With increasing temperature up to 100 °C, the *P*_r_ and *E*_c_ values decreased and the hysteresis loops started to deform and showed constricted loops for the 0–0.1 mol% Al_2_O_3_ samples. This indicates the onset temperature for ergodic behavior (see marked as “*”). However, the behavior of the constricted loops was not evident even at ∼100 °C for the 0.5–1.0 mol% Al_2_O_3_ samples. It is clearly suggested that the onset temperature for ergodic behavior was pushed to higher temperatures as Al_2_O_3_ content increased.^49,50^ While at a higher temperature of 150 °C, the *P*_r_ and *E*_c_ values rapidly decreased close to zero and the hysteresis loops for all compositions appeared with a characteristic double pinched shape. This observation supports our observation for the shift of the depolarization temperature to a higher temperature. This trend is apparently well correlated with the higher depolarization temperature as shown in Fig. 5, which was determined by the thermally stimulated depolarization current (TSDC). In addition, the decrease of *P*_r_ and *E*_c_ values with increasing temperature in Fig. 8(a) and (b) is consistent with that reported by Malik *et al.*^50^ in the 0.96[Bi_0.5_(Na_0.84_K_0.16_)_0.5_Ti_1−*x*_Ta_*x*_O_3_]–0.04SrTiO_3_ lead-free ceramics. Their result also showed that the *P*_r_ and *E*_c_ values decreased at high temperatures due to the chemical substitution and temperature effects. Both factors can cause lattice distortion and disrupt the FE long-range order leading to the decrease in the stability of the polarization.^50^ Gupta *et al.*^33^ also found that there was a sudden decrease in *P*_r_ and *E*_c_ values around the *T*_F–R_ (∼100 °C) for the binary system of (1 − *x*)BNKT–*x*SHZ, resulting in the observation of a pinched *P*–*E* loop, which is typical for ergodic relaxors (ER). In the work done by Patterson *et al.*,^51^ they investigated the solid solutions of BNT and Bi(Zn_0.5_Ti_0.5_)O_3_ (BZT) and they found that with 2 mol% of BZT, the double loops appeared at around 150 °C. Temperature-dependent hysteresis measurements also showed that the *P*_r_ and *E*_c_ values decreased significantly and the data showed double-loop characteristics on heating.

**Fig. 9:**
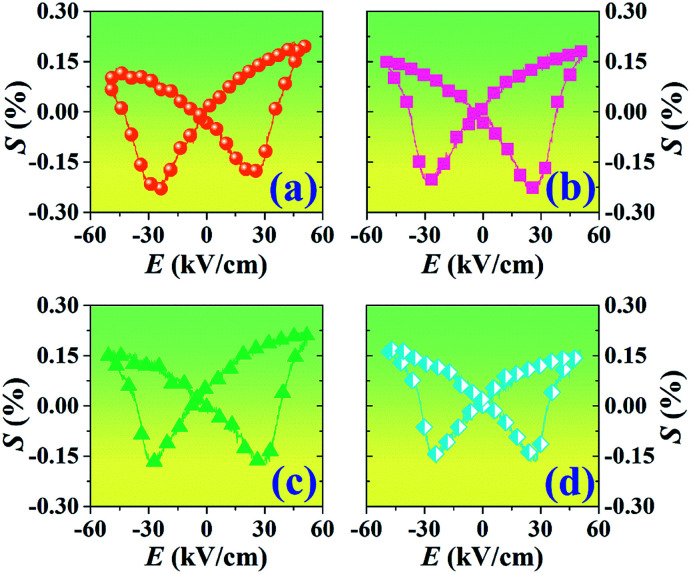
Bipolar strain–electric field (*S*–*E*) loops of the BNT–6BT/*x*Al_2_O_3_ ceramics where *x* = 0–1.0 mol%, measured under an electric field of 50 kV cm^−1^ and a frequency of 0.1 Hz.

Fig. 9 presents the bipolar strain–electric field (*S*–*E*) loops of the BNT–6BT/*x*Al_2_O_3_ ceramics measured at room temperature (RT). The measurement was performed under an electric field of 50 kV cm^−1^ and a frequency of 0.1 Hz. For more details, the maximum strain (*S*_max_), the negative strain (*S*_neg_, *i.e.* denotes the difference between zero-field strain and the lowest strain and is only visible in the bipolar cycle),^52^ and the normalized strain coefficient 
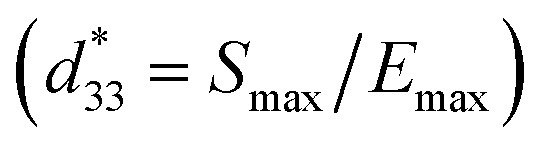
^3,52^ are also summarized in Table 2. The pure BNT–6BT ceramic exhibited a typical butterfly-shaped strain loop with *S*_max_ of 0.21% and 
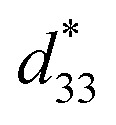
 of 412 pm V^−1^ where the largest *S*_neg_ of −0.22% was observed.^6^ The *S*_max_ and 
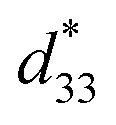
 value of this composition in this work is higher than the *S*_max_ (0.18%) and 
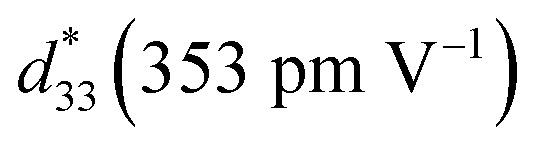
 values of the 0.94(Bi_0.5_Na_0.5_)TiO_3_–0.06BaTiO_3_ ceramic, observed in previous works.^3^ With increasing Al_2_O_3_ content in the BNT–6BT ceramic, the *S*_max_ and 
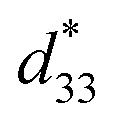
 values slightly increased to 0.23% and 466 pm V^−1^, respectively for the 0.1 mol% Al_2_O_3_ ceramic. With a further increase in the Al_2_O_3_ content, the *S*_max_ and 
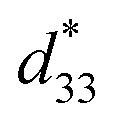
 values slightly decreased.

### Piezoelectric and energy harvesting properties

**Fig. 10:**
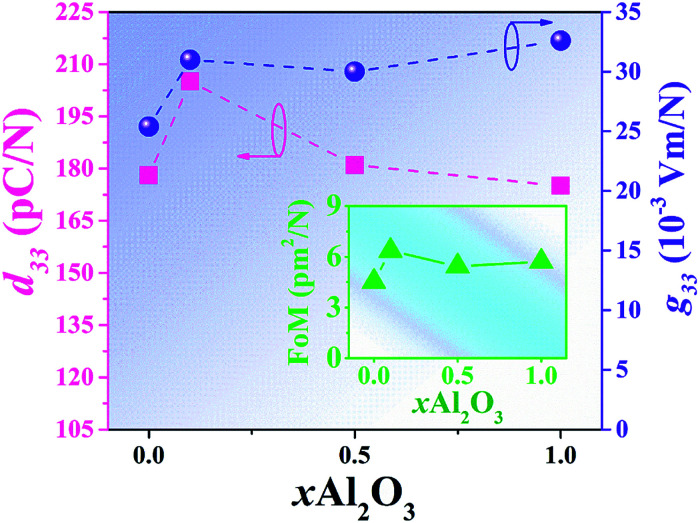
Plots of low-field piezoelectric coefficients (*d*_33_) and piezoelectric voltage coefficient (*g*_33_) as a function of Al_2_O_3_ content of the BNT–6BT/*x*Al_2_O_3_ ceramics (the inset shows: FoM as a function of Al_2_O_3_ content).

Plots of low-field piezoelectric coefficient (*d*_33_) as a function of Al_2_O_3_ nanoparticle content of the BNT–6BT/*x*Al_2_O_3_ ceramics are shown in Fig. 10 and their values are also summarized in Table 2. The pure BNBT ceramic had a low-field *d*_33_ of 178 pC N^−1^. The low-field *d*_33_ value increased with increasing Al_2_O_3_ content and reached a maximum value of 205 pC N^−1^ for the 0.1 mol% Al_2_O_3_ ceramic. This value was close to that presented by Yoo *et al.*^53^ in an earlier study, where the low-field *d*_33_ value of 205 pC N^−1^ for the BNT-based system was obtained. With a further increase in the Al_2_O_3_ content, the low-field *d*_33_ slightly decreased. The improvement of low-field *d*_33_ for the modified samples confirms the improvement of ferroelectric properties. This can be explained by the thermodynamic theory of ferroelectrics. According to this theory, *d*_33_ can be expressed as *d*_33_ = 2*ε*_33_^*T*^*Q*_11_*P*_r_, where *ε*_33_^*T*^ represents the dielectric constant of material, *Q*_11_ represents the electrostrictive coefficient, which is constant for perovskite materials, and *P*_r_ is the remnant polarization.^54^ This equation suggests that the *d*_33_ value is proportional to the *P*_r_ value. Therefore, the increase of the *d*_33_ value can be due to the improvement of the *P*_r_ value. Furthermore, the radius of Al^3+^ (0.535 Å) is closer to the radius of Ti^4+^ (0.605 Å). When Al^3+^ occupies the Bi-site of Ti^4+^ as an acceptor within the perovskite ABO_3_ structure,^48^
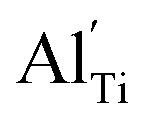
 has an effective negative charge, which can be compensated by a positively charged oxygen vacancy^55^ in order to maintain the overall charge neutrality.^15^ The oxygen vacancy can produce lattice deformation. This can make the ferroelectric domain reorientation easier during electrical poling, and then lead to the enhancement of piezoelectric properties.^19^ Moreover, the increase in low-field *d*_33_ can be linked to an increase in the grain size as it is known that the larger gain gives the larger domain size with smaller domain wall width, thus enabling domain movement (under an applied electric field).^20,56,57^

The piezoelectric voltage coefficient (*g*_33_) value is a key factor when evaluating piezoelectric energy harvester. It is a parameter that indicates the amount of electrical energy generated by the applied pressure.^58^ Normally, it is difficult to obtain high *g*_33_ for an increase in *d*_33_ usually accompanied by an even larger increase in *ε*_r_ value.^59^ The *g*_33_ value can be explained by the following relation:^58–62^

2 *g*_33_ = *d*_33_/*ε*_r_*ε*_0_

where *g*_33_ is the piezoelectric voltage constant, *ε*_r_ is the dielectric constant of the piezoelectric material, *ε*_0_ is the dielectric constant in a vacuum, and *d*_33_ is the piezoelectric charge constant. Plots of *g*_33_ as a function of Al_2_O_3_ content of the BNT–6BT/*x*Al_2_O_3_ ceramics are shown in Fig. 10. The *g*_33_ value was improved by the addition of Al_2_O_3_. The *g*_33_ value increased from 25.4 × 10^−3^ Vm N^−1^ for the pure BNT–6BT ceramic to the maximum value of 32.6 × 10^−3^ Vm N^−1^ for the 1.0 mol% Al_2_O_3_ ceramic. Normally, the *g*_33_ constant is proportional to the piezoelectric constant and inversely proportional to the permittivity.^62^ An improvement of the *g*_33_ value for this work is due to an improvement of the *d*_33_ and a lower *ε*_r_ value in this sample.

The off-resonance figure of merit (FoM) for energy harvesting can be expressed as follows:^58,62,63^

3 FoM (pm^2^ N^−1^) = *d*_33_ × *g*_33_

where *d*_33_ and *g*_33_ are the piezoelectric charge constant and the piezoelectric voltage constant of the device, respectively. The trend of the FoM of the BNT–6BT/*x*Al_2_O_3_ ceramics is similar to that of the *g*_33_ value which can be seen in Fig. 10. The pure BNT–6BT ceramic had the FoM value of 4.52 pm^2^ N^−1^. With increasing Al_2_O_3_ content, the FoM increased to 6.36 pm^2^ N^−1^ for the 0.1 mol% Al_2_O_3_ ceramic, which was more than 1.4 times as compared to the pure BNT–6BT, and then slightly decreased with a further increase in the Al_2_O_3_ content. The FoM value for this composition (0.1 mol%) is close to the FoM value of 5.46 pm^2^ N^−1^ for the 0.55BZT–0.45BCT ceramic sintered at 1475 °C, observed previously by Shin *et al.*^58^ Based on the obtained data, the Al_2_O_3_ additive produced an improvement of the energy harvesting behavior of this ceramic system. Therefore, Al_2_O_3_ doping may be a good way for the improvement of energy harvesting behavior for other BNBT-based ceramics. The highest FoM of 6.36 pm^2^ N^−1^ as observed for the 0.1 mol% Al_2_O_3_ ceramic, suggests that this ceramic has the potential to be one of the promising lead-free piezoelectric candidates for further use in energy harvesting applications.

## Conclusions

To sum it all up, the lead-free 0.94(Bi_0.5_Na_0.5_)TiO_3_–0.06BaTiO_3_/*x*Al_2_O_3_ or BNT–6BT/*x*Al_2_O_3_ ceramics (with *x* = 0, 0.1, 0.5, and 1.0 mol%) were successfully synthesized by a solid-state mixed oxide method. Phase, microstructure and electrical properties of the ceramics as a function of Al_2_O_3_ nanoparticle-added content were examined. The XRD and Raman spectra showed coexisting rhombohedral and tetragonal phases throughout the entire compositional range. The additive promoted grain growth and improved the electrical properties of the ceramics. The *T*_d_, *T*_F–R_ and *ε*_max_ values tended to increase with Al_2_O_3_ concentration. The 0.1 mol% Al_2_O_3_ ceramic showed good ferroelectric properties and relatively high piezoelectric coefficients. The off-resonance figure of merit (FoM) for energy harvesting was also improved by the Al_2_O_3_ additive. It is believed that these results can provide a simple and effective way to defer the thermal depolarization to a higher temperature and to improve the electrical properties and thermal stability of conventional (BNT–BT)-based and other (BNT–BT)-based ceramics, which may pave the way for further use for wide temperature applications.

## Conflicts of interest

There are no conflicts to declare.

## Supplementary Material

## References

[cit1] Yan B., Fan H., Wang C., Zhang M., Yadav A. K., Zheng X., Wang H., Du Z. (2020). Ceram. Int..

[cit2] Li Q., Ning L., Hu B., Peng H., Zhao N., Fan H. (2019). Ceram. Int..

[cit3] Wu L., Shen B., Hu Q., Chen J., Wang Y., Xia Y., Yin J., Liu Z. (2017). J. Am. Ceram. Soc..

[cit4] Bai W., Wang L., Zhao X., Zheng P., Wen F., Li L., Zhai J. (2019). Ceram. Int..

[cit5] Zhao N., Fan H., Ren X., Ma J., Bao J., Guo Y., Zhou Y. (2019). J. Eur. Ceram. Soc..

[cit6] Gong Y., He X., Chen C., Yi Z. (2019). Ceram. Int..

[cit7] Mohamad N. B., Shakarchi E. K. A. (2017). J. Adv. Dielectr..

[cit8] Nagata H., Shinya T., Hiruma Y., Takenaka T., Sakaguchis I., Haneda H. (2005). Ceram. Trans..

[cit9] Takenaka T., Maruyama K., Sakata K. (1991). Jpn. J. Appl. Phys..

[cit10] Li Q., Li M., Wang C., Zhang M., Fan H. (2019). Ceram. Int..

[cit11] Jaffe B. , Cook W. R. and Jaffe H., Piezoelectric Ceramics, Academic Press, London, 1971

[cit12] Zhang J., Pan Z., Guo F. F., Liu W. C., Ning H., Chen Y. B., Lu M. H., Yang B., Chen J., Zhang S. T., Xin X., Rodel J., Cao W., Chen Y. F. (2015). Nat. Commun..

[cit13] Yin J., Wang Y., Zhang Y., Wu B., Wu J. (2018). Acta Mater..

[cit14] Anton E. M., Jo W., Damjanovic D., Rödel J. (2011). J. Appl. Phys..

[cit15] Mahajan A., Zhang H., Wu J., Ramana E. V., Reece M. J., Yan H. (2017). J. Phys. Chem. C.

[cit16] Takagi Y., Miura T., Nagata H., Takenaka T. (2019). Jpn. J. Appl. Phys..

[cit17] Verma A., Yadav A. K., Kumar S., Srihari V., Rajput P., Reddy V. R., Jangir R., Poshwal H. K., Liu S. W., Biring S., Sen S. (2018). J. Appl. Phys..

[cit18] Li L., Fang D., Wang R. X., Hao J., Gu Z. B., Zhang S. T. (2020). Ceram. Int..

[cit19] Lee Y. C., Lee T. K., Jan J. H. (2011). J. Eur. Ceram. Soc..

[cit20] Coondoo I., Agarwal S. K., Jha A. K. (2009). Mater. Res. Bull..

[cit21] Hayati R., Barzegar A. (2010). Mater. Sci. Eng., B.

[cit22] Jaita P., Kruea-In C., Rujijanagul G. (2016). Nanomater. Nanotechnol..

[cit23] Kohn J. A., Katz G., Broder J. D. (1957). Am. Mineral..

[cit24] Cheng R., Xu Z., Chu R., Hao J., Du J., Ji W., Li G. (2015). Ceram. Int..

[cit25] Chen J., Wang Y., Wu L., Hu Q., Yang Y. (2019). J. Alloys Compd..

[cit26] Shannon R. D. (1976). Acta Crystallogr..

[cit27] Wang C., Li Q., Yadav A. K., Peng H., Fan H. (2019). J. Alloys Compd..

[cit28] Raju K., Kandula K. R., Asthana S., Patri T. (2019). Phase Transitions.

[cit29] Yadav A. K., Fan H., Yan B., Wang C., Zhang M., Ma J., Wang W., Dong W., Wang S. (2020). Ceram. Int..

[cit30] Baskar S., Meyricka D., Selvan R. K., Minakshi M. (2014). Chem. Eng. J..

[cit31] Baskar S., Selvan R. K., Vasylechko L., Minakshi M. (2014). Solid State Sci..

[cit32] Wang C., Li Q., Zhang W., Yan B., Yadav A. K., Peng H., Fan H. (2020). Ceram. Int..

[cit33] Gupta S. K., Quade R. M., Gibbons B., Mardilovich P., Cann D. P. (2020). J. Appl. Phys..

[cit34] Zhou X., Yana Z., Qi H., Wang L., Wang S., Wang Y., Jiang C., Luo H., Zhang D. (2019). J. Eur. Ceram. Soc..

[cit35] Basar N. U., Khan M. I., Ullah A., Ullah N., Kim I. W., Rehman N. U., Khan J. (2019). Mater. Res. Express.

[cit36] Bai W., Wang L., Zhao X., Zheng P., Wen F., Li L., Zhai J., Ji Z. (2019). Dalton Trans..

[cit37] Dong G., Fan H., Shi J., Li Q. (2018). J. Am. Ceram. Soc..

[cit38] Hussain A., Rahman J. U., Zaman A., Malik R. A., Kim J. S., Song T. K., Kim W. J., Kim M. H. (2014). Mater. Chem. Phys..

[cit39] Ke S., Fan H., Huang H., Chan H. L. W. (2008). Appl. Phys. Lett..

[cit40] Bokov A. A., Ye Z. G. (2000). Solid State Commun..

[cit41] Wang T., Chen X. M., Qiu Y. Z. (2017). Ferroelectrics.

[cit42] Lei N., Zhu M., Wang L., Yang P., Hou Y. (2011). Phys. Status Solidi A.

[cit43] Hu H., Zhu M., Xie F., Lei N., Chen J., Hou Y., Yan H. (2009). J. Am. Ceram. Soc..

[cit44] Li L., Zhu M., Zhou K., Wei Q., Zheng M., Hou Y. (2017). J. Appl. Phys..

[cit45] Mudinepalli V. R., Feng L., Lin W. C., Murty B. S. (2015). J. Adv. Ceram..

[cit46] Randall C. A., Kim N., Kucera J. P., Cao W., Shrout T. R. (1998). J. Am. Ceram. Soc..

[cit47] Li Q., Wang J., Ma Y., Ma L., Dong G., Fan H. (2016). J. Alloys Compd..

[cit48] Lee I. H., Lee H. S., Kim Y. H., Gil S. K., Kang D. H. (2013). Ceram. Int..

[cit49] Jaita P., Watcharapasorn A., Kumar N., Cann D. P., Jiansirisomboon S. (2015). Electron. Mater. Lett..

[cit50] Malik R. A., Hussain A., Maqbool A., Zaman A., Song T. K., Kim W. J., Kim M. H. (2016). J. Alloys Compd..

[cit51] Patterson E. A., Cann D. P. (2012). J. Am. Ceram. Soc..

[cit52] Malik R. A., Hussain A., Maqbool A., Zaman A., Ahn C. W., Rahman J. U., Song T. K., Kim W. J., Kim M. H. (2015). J. Am. Ceram. Soc..

[cit53] Yoo J., Hong J., Lee H., Jeong Y., Lee B., Song H., Kwon J. (2006). Sens. Actuators, A.

[cit54] Ullah A., Ahn C. W., Hussain A., Kim I. W., Hwang H. I., Cho N. K. (2010). Solid State Commun..

[cit55] Moulson A. J. and Herbert J. M., Electroceramics: Materials, Properties, Applications, John Wiley & Sons Ltd., Chichester, 2003

[cit56] Ramajo L. A., Taub J., Castro M. S. (2014). Mater. Res..

[cit57] Manotham S., Butnoi P., Jaita P., Tantraviwat D., Boothrawong N., Rujijanagul G. (2020). Mater. Res. Bull..

[cit58] Shin D. J., Kim J., Koh J. H. (2018). J. Eur. Ceram. Soc..

[cit59] Liu B., Li P., Shen B., Zhai J., Zhang Y., Li F., Liu X. (2018). J. Am. Ceram. Soc..

[cit60] Shin D. J., Koh J. H. (2016). J. Nanosci. Nanotechnol..

[cit61] Gowdhaman P., Annamalai V., Thakur O. P. (2016). Ferroelectrics.

[cit62] Choi Y. J., Yoo M. J., Kang H. W., Lee H. G., Han S. H., Nahm S. (2013). J. Electroceram..

[cit63] Shin D. J., Kang W. S., Koh J. H., Cho K. H., Seo C. E., Lee S. K. (2014). Phys. Status Solidi A.

